# Use of Sensors in the Treatment and Follow-up of Patients with Diabetes Mellitus

**DOI:** 10.3390/s100807404

**Published:** 2010-08-09

**Authors:** Isabel Torres, Maria G. Baena, Manuel Cayon, Jose Ortego-Rojo, Manuel Aguilar-Diosdado

**Affiliations:** Endocrinology and Nutrition Service, Hospital Puerta del Mar, Ana de Viya 21, 11009 Cadiz, Spain; E-Mails: isabeltoba@gmail.com (I.T.); gloria.baenanieto@gmail.com (M.G.B.); m.cayonblanco@terra.es (M.C.); joseortegorojo@gmail.com (J.O.-R.)

**Keywords:** diabetes mellitus, sensors, glucose control

## Abstract

Glucose control is the cornerstone of Diabetes Mellitus (DM) treatment. Although self-regulation using capillary glycemia (SRCG) still remains the best procedure in clinical practice, continuous glucose monitoring systems (CGM) offer the possibility of continuous and dynamic assessment of interstitial glucose concentration. CGM systems have the potential to improve glycemic control while decreasing the incidence of hypoglycemia but the efficiency, compared with SRCG, is still debated. CGM systems have the greatest potential value in patients with hypoglycemic unawareness and in controlling daily fluctuations in blood glucose. The implementation of continuous monitoring in the standard clinical setting has not yet been established but a new generation of open and close loop subcutaneous insulin infusion devices are emerging making insulin treatment and glycemic control more reliable.

## 1. Introduction

The term Diabetes Mellitus (DM) covers a heterogeneous group of diseases or metabolic syndromes characterized by hyperglycemia of different genotype-phenotypic characteristics and with multiple factors implicated in their etiology [1]. Worldwide, the prevalence of DM (essentially Type 2 DM) has increased progressively and alarmingly. Wild *et al.* [2] estimated in 2004 that the prevalence of DM for all age groups would increase from 171 million individuals in the year 2000 to 366 million by the year 2030. As a consequence, the rate of microvascular and macrovascular complications associated with the disease are expected to increase correspondingly [3] making DM a first-order socio-economic health-care problem with important repercussions on the quality-of-life (QoL), and of health-care costs.

Several prospective studies have demonstrated the tight association existing between glycemia and the development of micro- and macroangiopathy complications. In Type 1 DM, the most significant has been the Diabetes Control and Complications Trial (DCCT) [4]. It demonstrated that intensive treatment was accompanied by a reduction in the appearance and progression of the microvascular complications. Subsequent analyses of this population demonstrated that the beneficial effect persisted subsequent to the intervention, and that the beneficial effects extended to adverse cardiovascular events, as well [5]. With respect to Type 2 DM, the United Kingdom Prospective Diabetes Study (UKPDS) and the study by Kumamoto [6,7] demonstrated a decrease in microvascular complications with intensive therapy. As in Type 1 DM, the post-intervention follow-up showed a persistent benefit of glycemic control with respect to the appearance and progression of the micro- and macrovascular complications [8]. Despite these results, there continues to be considerable debate on the effect of strict glycemic control on the development of cardiovascular disease. Several large studies provide evidence regarding the impact of intensive glycemic control on cardiovascular complications, including the Action to Control Cardiovascular Risk in Diabetes (ACCORD) study, the Diabetes and Vascular Disease, Preterax and Diamicron Modified Release Controlled Evaluation (ADVANCE) study, and the Veterans Affairs Diabetes Trial (VADT) [9–11]. None of them found a significant benefit of aggressive glucose lowering on cardiovascular events.

As such, currently there is evidence available to be able to affirm that better glycemic control reduces the microvascular complications associated with DM. The current consensus continues to recommend an intensive approach to glycemic control, with an objective of HbA1c of between 6.5–7%. Taking into account the latest studies published, these objectives should be individualized, above all in patients with a long-term clinical evolution of diabetes, high risk of hypoglycemia, co-morbidities and history of cardiovascular disease.

To achieve this objective, treatment of DM has been continually enriched by several novel therapeutic approaches such that, currently, there is a wide therapeutic arsenal that has increased with the incorporation of insulin analogues [12–14] and the activators of the incretin effect [15–17]. Also, the introduction and optimization of the devices for the administration of the insulin (continuous infusion system) achieves profiles of activity closer to the physiological equivalent [18,19].

All patients with DM who receive treatment with insulin, and certain patients treated with oral hypoglycemic agents that can cause hypoglycemia (essentially secretagogue drugs), need to have regular checks of glucose concentrations so as to maintain an appropriate metabolic control. From the appearance of the technique (SRCG) towards the end of the 1970s, especially following the publication of the results of the DCCT, this method has experienced an enormous increase in its diffusion, use and technological development. The measurement of glucose using glucometers has passed from using a technique of photometric reflectance to a method based on an electrochemical biosensor. The modern enzymatic methods (hexokinase or glucose oxidase) provide rapid measurements that are reliable and precise [20].

Until the previous decade, the SRCG system has been the only method available to monitor levels of glycemia. The SRCG is a well developed technique, albeit invasive, and on many occasions, tedious for the patient. Further, the information device is intermittent and partial. Since it frequently does not retain the changes in glycemia that occur over the length of the day, the objectives sought are not achieved [21]. Recent clinical evidence has demonstrated that the glycemic profile of the patients with DM, especially Type 1 DM, is characterized by wide fluctuations related to physical activity, diet, and the specific treatment. The impact and the consequences of these variations are only partially known. Hence, the possibility to have continuous information available on the levels of glucose implies an attractive option [22].

From the U.S. FDA (Food and Drug Administration) approval of the ambulatory use of the first systems of continuous monitoring of glucose (glucose sensors) in the year 1999, their use has provided detailed information that has improved knowledge of diabetes useful in establishing measures of optimization of the different types of treatment [21,23]. This review centers on the use of sensors and monitoring treatment of patients with DM. It focuses on the types and clinical indications of the systems of continuous glucose monitoring, their advantages and disadvantages compared to self-monitoring of capillary blood glucose. Finally, it analyzes the utility of the systems of continuous subcutaneous insulin infusion, and suggests some approaches for the future.

## 2. Continuous Monitoring of Glucose: Glucose Sensors

Glucose sensors, or continuous glucose monitoring systems (CGMS) provide the maximum information on the modifications of the plasma glucose levels along the course of the day and can be used in optimizing treatment in patients with DM [24]. The CGMS enables the oscillations in glycemia to be detected in a continuous manner and this provides information on the direction, magnitude, duration, frequency and possible causes of changes in the glucose levels. This overcomes the limitations of the information provided by the SRCG system in intensive treatment, above all in cases of inadvertent hypo- and hyperglycemias [21]. However, the sensors currently available do not have the precision of the capillary glucometers. This limitation is due, in part, to the low concentration of glucose in the interstitial fluid, the specific dynamics of glucose, and the delays inherent in the system of measurement. Further, the patients who use CGMS are obliged to perform frequent SRCG since the sensors need to be calibrated. For these reasons, SRCG continues to be recommended for therapeutic decisions making and CGMS is currently approved only as a complementary technique to the capillary measurement of glycemia [25].

### 2.1. The functioning of glucose sensors: Types of systems for the continuous monitoring of glucose

Continuous monitoring of glucose was introduced in the decade of the 1970s with a complex system termed biostator, or artificial pancreas. Subsequently, new generation sensors appeared, with variable results. The initial non-invasive systems that had been commercialized *i.e.*, GlucoWatch G2 Biographer® (Cygnus, Redwood City, CA, USA) and Pendra® (Pendragon Medical, Zurich, Switzerland), had been withdrawn because of, among other reasons, the lack of precision. Currently there are four types of minimally invasive CGMS approved by the FDA and are available on the international market. These are the CGMS® (Continuous Glucose Monitoring System) such as the Guardian®, the Guardian Real Time®, the Paradigm Real Time® (Medtronic MiniMed, Northridge, CA, USA); the GlucoDay® system (Menarini Diagnostics, Florence, Italy); the Seven System® (DexCom Inc., San Diego, CA, USA); and the Freestyle Navigator® monitor (Abbott Laboratories, Alameda, CA, USA) [26].

The CGMS currently available continuously measure the concentration of glucose in the interstitial fluid of the subcutaneous tissue using an amperimetric sensor linked to an electro-chemical enzymatic process. The electrons generated are quantified by the process of glucose oxidation by the enzyme glucose oxidase; the current generated is directly proportional to the glucose concentration. These systems are based on the premise that the concentration of glucose in the interstitial fluid correlates well with the plasma glucose due to its diffusion across the wall of the capillaries. The system is defined as minimally invasive because it requires the insertion into the subcutaneous cellular tissue, generally in the abdomen, of a fine needle to measure the glucose in the interstitial fluid directly, or via an external sensor; the signal generated is collected and processed in an external monitor. The CGMS®, Guardian®, Guardian Real Time®, Paradigm Real Time®, Seven System® and Freestyle Navigator® systems are *in situ* sensors and only the GlucoDay® system has an external sensor [19,26]. Table 1 summarizes the basic characteristics of the different sensors currently available.

Following an initial period of variable latency, and prior calibration with the SRCG, the monitor determines glucose concentration every 1–10 minutes over a period of between 2 and 7 days. The results can be obtained in real time or retrospectively, depending on the type of sensor [19,27]. With systems with real time reading of results, the data are generated after a period of initial latency and of the first calibration. However, there is a period of delay of up to 1.5 minutes, depending on the type of system, between the measurement of the glucose in the interstitial fluid and the generation of the result by the monitor. The current CGMS have alarms for the hypoglycemia and hyperglycemia episodes and some models provide a summary of the trends of the glucose measurements. In situations of euglycemia, the mean of the differences of each new value of capillary-interstitial glycemia is around 16 mg/dL (0.9 m mol/L) which implies a relative difference of 15%. The estimated clinical precision using point & rate error grid analysis varies between 75 and 88%, depending on the CGMS used [28,29]. With the systems that provide result retrospectively, the data are downloaded at the end of the registry using all the calibration points for adjustment. The correlation with the values of capillary glycemia is round 80% and the clinical precision estimated with the point & rate error grid analysis is around 70% [30–32]

#### CGMS, Guardian, Guardian RT and Paradigm RT

The CGMS approved by the FDA in 1999 was the first new generation sensor commercialized, and is the system that has been most widely used. The measurements are performed every 5 minutes over a maximum time of 72 hours. The sensor is connected via a cable to an external monitor which records the signals from the sensor every 15 minutes. The results are not visualized in real time, but can be downloaded into a computer for their subsequent analyses. The device needs to be calibrated a minimum of four times a day using measurements of capillary glycemia. The dysphase between the level of glycemia and the signal from the sensor which corresponds to the concentration of glucose in the interstitial fluid is 4 minutes. Of note is the disadvantage of limited reproducibility and reliability of measured values below 50 mg/dL [33].

The system is a clear example of improvement and development of software and of the manufacturing process of glucose electrodes, and shows a considerable advance in sensor design from one generation to the next [34]. For example, in the year 2004, the FDA approved the Guardian® monitor, a version of a previous, technologically improved sensor that included a system of alarm not only for hyperglycemia but also for hypoglycemia. One year later Guardian Real Time® appeared which enabled the measurement of glucose to be read in real time, and every 5 minutes, following a period of 2 hours of latency. The Paradigm Real Time® was approved in the year 2006 as CGMS combined with a continuous subcutaneous insulin infusion (CSII) device. More recently, the MiniMed Paradigm® Veo System has been introduced. It includes a CSII with a CGMS and is equipped with a Low Glucose Suspend (LGS) mechanism, which stops insulin delivery automatically whenever glucose levels are too low. All these systems required a minimum of 2 calibrations a day, and which is done by introducing the capillary glycemia value into the monitor [26].

#### GlucoDay

The GlucoDay system has been available on the market since 2002. Its method of function is based on the technique of micro-dialysis applied to the interstitial fluid which, as has been commented upon earlier, enables the measurement of the glucose concentration in the subcutaneous tissue [35,36]. This system is composed of a catheter for the micro-dialysis that is inserted subcutaneously, generally in the peri-umbilical region, and is connected to an external portable unit. An isotonic fluid without glucose is perfused such that the glucose of the interstitial fluid surrounds the inserted catheter depending on the concentration gradient. The level of glucose in the dialyzed liquid is determined each second and a mean value is stored every 3 minutes. The result is a total of 480 measurements per day. The maximum interval of continuous measurement is 48 hours. The data collected by the sensor can be visualized in real time. They are used subsequently to analyze the changes in the concentration of glucose and can be viewed graphically. The dysphase between the values of the subcutaneous glucose and the venous plasma is >3 minutes [24,37]. The system shows high precision and reliability even in low levels of glucose [24].

#### Seven System

This system, approved by the FDA in 2007, enables real time reading of the measurements of glucose performed every 5 minutes following a latency period of 2 hours, over a maximum period of 7 days. Also, the results of the glucose measurements can be collected for subsequent analyses. The system includes alarms that can be programmed not only for hyperglycemia but also for hypoglycemia, and needs to be calibrated with values derived from capillary glycemia measurements every 12 hours. There is a new version of this system recently commercialized termed the Seven Plus System®. It has been improved not only in precision but also in functioning which includes numerous additional functions [26,38].

#### FreeStyle Navigator

After several years of meticulous assessment, the FDA approved this system in 2008. The system is based on the *Wired Enzyme®* technology which uses measurement not dependent on oxygen, in place of the peroxide of hydrogen used in the majority of the other devices. [38] It allows real time reading glucose determinations after a 10 hour latency period in the earlier versions of the systems available, and after 1 hour for the latest systems that have become available in some countries. The concentration of glucose is updated every minute and stored for retrospective analyses every 10 minutes. It requires calibration with at least 4–5 capillary glucose measurements from the start of the monitoring period. Also, systems of alarms are available for altered levels of glucose, and information is provided on the trends in levels [26].

### 2.2. Differences between self-monitoring of capillary glycemia and the continuous glucose monitoring system

The two types of techniques of glucose measurement differ in several aspects [22] (Table 2): (1) The intermittent measurement of capillary glucose using SRCG provides a limited number of very exact values, while the continuous monitoring provides multiple measurements of glucose, albeit less precise; (2) The results of the SRCG indicate the metabolic state present at the moment of performing the measurement but does not contain information as to the previous status or on the subsequent metabolic status, until a new measurement is made; (3) The SRCG does not predict future levels of glycemia, while with the CGMS one can observe tendencies and, as such, the system has a predicative capacity; (4) The conduct of SRCG sometimes is not compatible with the current activity of the patient such as, for example, during sleep.

### 2.3. Clinical indications

The clinical applications of the CGMS have not, as yet, been well established. This system can be especially useful in determining patterns of glycemia over 24 hours, and in detecting hypoglycemia, particularly that occurring at night and inadvertently. However, its capacity to improve metabolic control has been demonstrated only in certain clinical situations [39,40].

Currently there are studies and preliminary data on the possible uses; one of the most studied being the detection of asymptomatic hypoglycemia. There is an elevated frequency of asymptomatic hypoglycemia, especially during the night and, above all, in patients with Type 1 DM, detected with CGMS; the frequencies being underestimated with SRCG [27,39,40].

CGMS also enables the values of glucose to be expressed in a novel manner. The mean values can change rapidly with a new treatment, and it is not always practical to wait months to evaluate the levels of HbA1c. Continuous monitoring can show the time during which the patient remains within a specific range of glycemia, that which can be more useful than a single point of integration of data, as in the case of an isolated HbA1c value. The extended presentation of mean values of glycemia may be preferred in some patients to the presence of frequent peaks of hypoglycemia and hyperglycemia that can be compensated-for between yes and no in altering the level of HbA1c. The CGMS can detect these oscillations, as well as measure its amplitude; this index of variability of the glycemia providing information on the trends of the mean levels [22].

**Figure 1.**
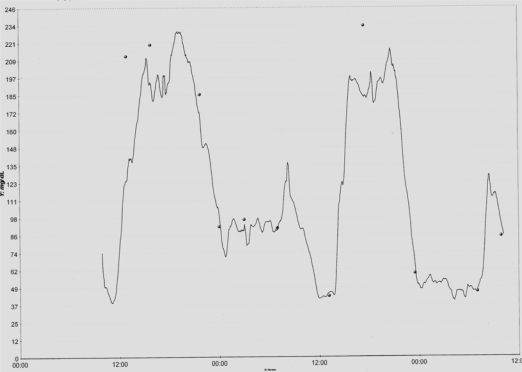
Representative graph of the glucose values obtained using the GlucoDay system in a patient with Type 1 DM. Note the wide variations in glycemia values, and the nocturnal hypoglycemia.

There has been observed in some studies an important correlation between markers of endothelial damage (oxidative stress and free radicals) and the oscillation of glycemia; the correlation being better than that observed with the levels of HbA1c [41]. Apart from providing information on the patterns of glycemia in the course of the day, the CGMS can characterize the modifications in the glycemic profile that are produced in certain periods such as, for example, in the postprandial period [40]. Figure 1 shows an example of the graphic representation of glucose values obtained using CGMS.

Some studies have demonstrated that the usefulness of CGMS, above all those that provide real-time data, resides in its capacity to modify treatment and to achieve improvement in metabolic control [39,40,42]. A study published recently demonstrated a clear benefit in the use of CGMS every 4 weeks in pregnant women with Type 1 and Type 2 DM in relation to the reduction in the levels of HbA1c and lower incidence of macrosomia. However, no reduction in hypoglycemia episodes was achieved, nor in Caesarian deliveries [43]. In relation to the real-time reading systems, 4 randomized studies have been published that demonstrated that the use of this type of sensor can improve the levels of HbA1c in children and adults with poor glycemic control [44]. A decrease in the concentrations of HbA1c is achieved when it is used ≥3–4 days per week [19]. Another study had observed that the use of the CGMS could decrease hypoglycemia and hyperglycemia episodes, as well as nocturnal hypoglycemia; the patients are within the range of objective of glycemic control for longer [45]. In the publication with the greatest number of patients in the series, 322 patients with sub-optimum metabolic control were treated not only with multiple doses of insulin but also with CSII. The results showed a decrease in the levels of HbA1c of 0.5% in adults. However, this reduction was not observed in children, adolescents and young adults and there were no differences in the number of severe hypoglycemic episodes [57].

Recently the American Diabetic Association (ADA) established the recommendations for the use of the CGMS based on the scientific evidence [25]: (1) CGMS, together with intensive insulin treatment can be a useful tool in further reducing the levels of HbA1c in selected adult patients (>25 years of age) with Type 1 DM (evidence grade A); (2) Although the evidence of a decrease in the levels of HbA1c is less in children, adolescents and young adults with DM, the CGMS can be useful in these patients as well; its benefits correlating well with the degree of use of the systems (evidence level C); (3) The CGMS can become a complementary tool in patients with unnoticed hypoglycemias and/or frequent episodes of hypoglycemia (evidence level E).

As such, the indications for CGMS apply to: those situations that require detailed information on the fluctuations of the glycemia; of diagnosis and management of hypoglycemias that are, essentially, inadvertent and nocturnal; evaluation of the response to new therapeutic plans; evaluation of the impact of the modification of life-style on the control of glycemia; monitoring situations which require a strict glycemic control in the absence of hypoglycemia.

In our experience, in a series of 545 patients with Type 1 DM having difficulty achieving appropriate metabolic control with multiple doses of insulin, we used CGMS with the GlucoDay® system to evaluate the fluctuations of glycemia and the frequency of hypoglycemia. The evaluation was, among other reasons, in order to be able to make adjustments to the treatment with multiple doses of insulin, and to change the treatment to CSII in 20 patients. Patients treated with CSII for at least 3 months continued to performing CGMS, the results from which were used to evaluate the response to treatment. With CSII, there was a significant decrease in the time of hypoglycemia during the dawn period (04:00 to 08:00 h), as well as a reduction in the area under the curve for hyperglycemia during the 24 h of monitoring and of the area under the cure of hyperglycemia during the period before dinner. This was accompanied by a significant decrease in the levels of HbA1c, without any differences being observed in the monitoring of the episodes of hypoglycemia [46].

### 2.4. Inconveniences

The major problems with CGMS are: a limited level of precision for the measurement of isolated glucose, especially at low levels; the need for several calibrations per day using capillary glucose controls; the short period of use (between 2 and 7 days) [27,38].

The minimally invasive CGMS can have secondary effects which are generally light and related to the local irritation in the zone of the catheter inserted for the continuous measurement of the interstitial fluid. Further, because of the complexity of the technique, these systems require the help of a professional health-care team on permanent stand-by [38].

Real time data are not only an important advance in patient self-care but also involves the safety and rigor for this technology. One important limitation is that none of the sensors currently available have the precision of the usual glucometers because they do not directly measure the glucose in the blood but, instead, are extrapolations from the glucose in the subcutaneous interstitial fluid. The defects of precision are related to the low concentrations of glucose in the interstitial fluid, the dynamics of the glucose itself in the capillary fluid and interstitial fluid, and the delays inherent in the system of measurement *i.e.*, variations in the interstitial fluid involves a delay with respect to the changes in the blood and this would vary according to the level of glycemia as well as on the device used.

The major limitation of the CGMS is the current absence of scientific evidence regarding its usefulness. As such, methodologically appropriate clinical studies are needed to evaluate the impact of these systems on the grade of control of Hba1c and other metabolic variables, such as the frequency of hypoglycemias, glycemic variability, time of exposure to hyperglycemia and on the QoL. This would enable decisions to be made regarding cost-effectiveness of CGMS use, and towards establishing its clinical indications more firmly.

## 3. Continuous Glucose Monitoring and Subcutaneous Insulin Infusion Systems

There are two effective methods of intensive therapy available: treatment with multiple subcutaneous injections of insulin, and the use of CSII systems. Several studies have demonstrated that the treatment with CSII improves the glycemic variation. Stricter glycemic control is achieved with greater decrease in the HbA1c, less hypoglycemias, and no increase in the frequency of ketoacidosis [47–49]. Therapy with CSII is recommended when a good metabolic control is not achieved with multiple doses of insulin, and in patients motivated by a greater flexibility in their life-style, provided they have been trained in the knowledge and necessary aptitude, and have sufficient health-care support available [50–52].

The usual indications are summarized in Table 3. The appropriate selection of the patients is fundamental in improving the outcomes and to minimize the risks; a highly important factor being the frequent monitoring of the glycemia every day. An interesting application of the CGMS in this situation is for initiating or adjusting the treatment with CSII. The information provided by the CGMS enables a better rhythm of basal infusion of insulin as well as the bolus injection. If, as it is acknowledged, that patients treated with CSII are more motivated, then effectiveness of the information derived from CGMS in this group could be even greater [42,53].

Currently, patients treated with CSII can use *in situ* subcutaneous sensors with real time readers which integrate the CGMS into a continuous infuser of insulin via cordless communication [54]. In this system the monitor of the sensor is located technically just inside the monitor of the infuser, and glucose levels detected by the GCMS system can be evaluated in real time. Also, historical values are registered in the hours before, and predictive information is generated on the trend towards increase or decrease in the glycemia. Further, these systems, as do regular sensors, have alarms (sound or vibration) for inappropriate high or low levels of glucose which can be programmed according to the metabolic objectives established in each patient. In this manner the CSII user can adapt the infusion of insulin and diet to the real metabolic status given that the data generated by the CGMS detects inadvertent hypoglycemias, informs on the trends, and on the speed of change of the glucose levels, and helps to plan the bolus to reduce the time of hyperglycemia [54]. A recent innovation has consisted of semi-automatic models that, in addition to integrating the infusion of insulin and the CGMS, have an automatic device incorporated that delays the infusion of insulin over 2 hours in case of an alarm indicating hypoglycemia that had been a response on the part of the user. Also, these systems have tools to download the information to the computer (capillary glycemia, interstitial glycemia, infuser data) and to generate graphical representation of the data. The retrospective analysis enables a pattern to be identified and the visualization of problems (variations in the glycemia over the length of the day, postprandial hyperglycemias, bolus injections not performed, over-reaction in hypoglycemias, prolonged disconnections, *etc*.). These serve to help in therapeutic adjustments, and are good tools for improving the patient’s education, adherence to therapy, and motivation [39].

Also, the models of CGMS integrated into insulin infusers require calibration using the simultaneous measurements of glucose in capillary blood. Capillary blood varies considerably with time. To obtain optimum calibration outcomes it is fundamental that the patient performs this operation at the times of the day in which the glucose levels are most stable, and to avoid calibrating when the glycemia is in a state of change. Hence, although the patient may be disposed to CGMS devices integrated with his insulin infuser, there is a need to continue performing the traditional self-analysis and to consider that the CGMS only as a complement to the measurement of capillary glucose; albeit more technologically developed and dynamic [54]. Nevertheless, as with patients treated with CSII combined with real time CGMS, the capillary self-analysis is needed to confirm the measurement generated by the CGMS under certain conditions *i.e.,* when the data from the CGMS are not in agreement with the sensations of the patients, before the administration of an insulin bolus, or in response to the alarms, in calibrating the system, confirming the response to treatment before an episode of hypoglycemia, and before driving motorized vehicles.

The candidate patients for CSII and CGMS need to be very motivated and to know that one of the most critical determinants for the benefit of this technology is the continuity of the use of the sensor which, in practice, can be the difficult to fulfill [39,55]. The patient needs to receive verbal and written information in a program of therapeutic education designed to avoid false expectation and to recognize the limitations of these systems. The education program will need to be orientated to the training in the appropriate management of the devices and in the interpretation of the measurements of glucose in real time such that the updates will be correct, and orientated towards the achievement of euglycemia [54].

The design and execution of appropriate clinical studies to evaluate the efficacy of the therapy with CSII combined with real time CGMS are complicated and can include many confounding factors. This technology is not amenable to the tradition double blind, controlled clinical trials that are applied in evaluating, for example, new drugs and devices. Also, the results will always be dependent on the level of training of the patients and their suitable reaction to the concentrations of glucose detected by the system. Several studies have shown that the use of CGMS in patients with CSII have advantages in metabolic control, with significant decreases in the levels of HbA1c related to adherence to treatment and to the frequency of use of the CGMS; continuous use being more efficient than intermittent [53,56,57]. Some studies have demonstrated improvement in the frequency of hypoglycemias, as well [42,54]. More studies are needed to establish the efficacy of this technology over the long term as well as the adaptability of the patients to the technology and the impact on the incidence of hypoglycemias, above all.

Finally, of note is that the therapy using CSII and CGMS is expensive. Implementing these technologies requires the Health-care Authorities to make available sufficient resources to initiate the treatment and to guarantee an appropriate follow-up.

## 4. Future Perspectives

The impact of CGMS in the management of patients with DM is increasing. The availability of CGMS systems integrated into CSII devices has produced a promising advance in the development of applications of biotechnology that improve the control of glycemia of the DM individuals. The closed loop system would include a continuous glucose sensor, an insulin infuser, and an algorithm of control that enables adjustments to be made of the infused insulin according to the concentrations of glucose measured [58]. However, although currently there are advanced insulin infusers available, there have not been glucose sensors developed that are continuous, reliable, and of long duration. On the other hand, to design an algorithm that combines the administration of insulin and the CGMS is complex and currently constitutes a field of intense research. These intelligent tools of analysis need to imitate the pancreatic physiology and to take into account not only the absolute values of glucose but also as well the changes in the glycemia over time, the patterns of response to food intake and to exercise, and the glycemic variations that occur during night-time sleep. When this technology is developed, the combined use of insulin infusers and glucose sensors can be converted into a strategy that is cost-effective and safe, with minimum limitations and an optimization of metabolic control of the diabetic patients in order to achieve glycemia that is very close to normality [19,59].

## Figures and Tables

**Table 1.** Main features of the currently available CGMS devices.

	**Guardian, Guardian RT Paradigm RT**	**GlucoDay**	**FreeStyle Navigator**	**Seven System**

Range of glucose values (mg/dL)	40–400	40–400	20–500	40–400
Life span of sensor (days)	3 in USA. / 6 in Europe	2	5	7
Warm-up period (hours)	2	2	10 (1 for latest system)	2
Calibration frequency	every 12 h	one point	Post-insertion: -10h, 12 h, 24h, 72h -1h, 2h, 10h, 24h, 72 h (latest system)	every 12h
Sensor site	*in situ*	External	*in situ*	*in situ*
Sensor device	Amperometric sensor glucose oxidase	Microdialysis glucose oxidase	Amperometric sensor Wired Enzyme®	Amperometric sensor glucose oxidase
Time results	Retrospective (Guardian) Real time (Guardian RT and Paradigm RT)	Retrospective and real time	Real time	Real time
Frequency of blood glucose display (min)	5	3	1	5
Rate-of-change arrows	Yes	No	Yes	Yes
Integrate with pump	Yes (Paradigm RT)	No	No	No
Accuracy (error grid) (%)_(24,28, 30–32)_	61.7– 76.3	64–88	76.3–81.7	70.4
Limitations _(19, 26)_	- Life span of 3 days (USA.) - Update glycemic data on the screen every 5 minutes. - Calibrations are required every 2–3 days.	- Large system - Life span of 2 days - Skin irritation - No rate-of-change arrows.	- Large sensor and transmitter. - Warm-up period of 10 hours (first sensor). - Calibration time programming required. - Must use Freestyle strips for calibration. - High cost.	-Update glycemic data on the screen every 5 minutes. -Does not permit selection of specific points above.

**Table 2.** Differential characteristics of the SRCG and CGMS.

	**SRCG**	**CGMS**
Precision	+++	++
Prompt information	+++	+
Overall information	+	+++
Patient initiative	++	+
Discomfort	+	++
Usefulness for the patient	+++	+
Evidence of effectiveness in glycemic control	+++	+

+ / +++: consensus agreement, or intensity.

**Table 3.** Indications for the insertion of the CSII device.

Inadequate metabolic control (HbA1c ≥7%) despite intensive multi-dose treatment and collaboration by the patient Planning for pregnancy, or during gestation, if the glycemic control is inadequate with other forms of intensive treatment Unstable (brittle) diabetes Inadvertent hypoglycemic episodes, frequent or severe Fasting hyperglycemia Variable daily schedule (work hours, meal times, travel *etc.*)
